# Compound heterozygous variants in *LAMC3* in association with posterior periventricular nodular heterotopia

**DOI:** 10.1186/s12920-021-00911-4

**Published:** 2021-02-27

**Authors:** Carla De Angelis, Alicia B. Byrne, Rebecca Morrow, Jinghua Feng, Thuong Ha, Paul Wang, Andreas W. Schreiber, Milena Babic, Ajay Taranath, Nick Manton, Sarah L. King-Smith, Quenten Schwarz, Peer Arts, Hamish S. Scott, Christopher Barnett

**Affiliations:** 1 Paediatric and Reproductive Genetics Unit, Women’s and Children’s Hospital, North Adelaide, SA Australia; 2 Genetics and Molecular Pathology Research Laboratory, Centre for Cancer Biology, An Alliance Between SA Pathology and the University of South Australia, Adelaide, Australia; 3 ACRF Cancer Genomics Facility, Centre for Cancer Biology, An Alliance Between SA Pathology and the University of South Australia, Adelaide, SA Australia; 4 South Australian Medical Imaging, Women’s and Children’s Hospital, North Adelaide, SA Australia; 5 Department of Surgical Pathology, Women’s and Children’s Hospital/SA Pathology, North Adelaide, SA Australia; 6 Neurovascular Research Laboratory, Centre for Cancer Biology, An Alliance Between SA Pathology and the University of South Australia, Adelaide, Australia; 7 School of Medicine, University of Adelaide, Adelaide, SA Australia; 8 School of Pharmacy and Medical Sciences, University of South Australia, Adelaide, SA Australia; 9 Australian Genomic Health Alliance, Melbourne, VIC Australia; 10 School of Biological Sciences, University of Adelaide, Adelaide, SA Australia; 11 SA Clinical Genetics Service, Women’s and Children’s Hospital, 72 King William Road, North Adelaide, SA 5006 Australia

**Keywords:** LAMC3, Periventricular nodular heterotopia, Occipital, Lobe, Case report

## Abstract

**Background:**

Periventricular nodular heterotopia (PNH) is a malformation of cortical development characterized by nodules of abnormally migrated neurons. The cause of posteriorly placed PNH is not well characterised and we present a case that provides insights into the cause of posterior PNH.

**Case presentation:**

We report a fetus with extensive posterior PNH in association with biallelic variants in *LAMC3*. *LAMC3* mutations have previously been shown to cause polymicrogyria and pachygyria in the occipital cortex, but not PNH. The occipital location of PNH in our case and the proposed function of LAMC3 in cortical development suggest that the identified *LAMC3* variants may be causal of PNH in this fetus.

**Conclusion:**

We hypothesise that this finding extends the cortical phenotype associated with LAMC3 and provides valuable insight into genetic cause of posterior PNH.

**Supplementary Information:**

The online version contains supplementary material available at 10.1186/s12920-021-00911-4.

## Background

Malformations of cortical development (MCD) are a group of disorders encompassing macroscopic and microscopic abnormalities of the cerebral cortex that have arisen during prenatal development [1]. The spectrum of MCD includes periventricular nodular heterotopia (PNH), where nodules of mis-localised neurons are abnormally arrested in their migration to the developing cerebral cortex. These ectopic neurons instead collect in the periventricular white matter, the location of the embryonic ventricular zone (VZ) [1, 2]. Peak neuronal migration occurs predominantly between the 12th to 24th weeks of gestation, meaning PNH can potentially be identified on ultrasound and magnetic resonance imaging (MRI) in pregnancy, but is difficult to diagnose before the third trimester [3].

Classical bilateral, symmetric PNH is associated with mutations affecting *FLNA* at Xq28 [4]. In the majority of individuals with PNH secondary to *FLNA* mutations, the nodules are located in the anterior bodies and frontal horns of the lateral ventricles (fronto-parietal) [4]. Individuals with classical PNH are typically female, and usually present with seizures and normal to borderline intelligence [4]. In addition to classical PNH, multiple chromosomal abnormalities as well as variants affecting 9 genes (*ARF1*, *ARFGEF2*, *DCHS1*, *ERMARD*, *FAT4*, *INTS8*, *MAP1B*, *MCPH1*, and *NEDD4L*) have been linked to PNH, indicating the genetic heterogeneity of the disorder [5–13].

Considerable heterogeneity is also observed in clinical presentation, with multiple subtypes of PNH having been described [2]. Bilateral posterior PNH, involving the occipital cortex, accounts for ~ 25% of all PNH cases, and differs from fronto-parietal, FLNA-associated PNH not only in the location of the nodules, but also in the increased likelihood of associated cortical malformations [14]. The genetic cause of many posterior PNH cases remains unknown, with mutations in only 3 genes (*ARFGEF2, ERMARD* and *NEDD4L*) being implicated as causal [6, 8, 12].

Here we report a male fetus with a severe presentation of isolated posterior PNH in which we identified compound heterozygous missense variants affecting the *LAMC3* gene (MIM 604349), which encodes the laminin subunit ɣ3. To our knowledge, this is the earliest identification of PNH by morphology ultrasound and the first report of *LAMC3* variants in a case of posterior PNH*.* Since mutations in *LAMC3* have previously been associated with other occipital MCD, it was an ideal candidate for the abnormalities identified in this fetus.

## Case presentation

### Study subjects

Written informed consent was provided by both parents for inclusion in the the Genomic Autopsy Study, an National Health and Medical Research Council (NHMRC) funded trio exome research study, approved by the Women's and Children's Health Network Human Research Ethics Committee (HREC/15/WCHN/35). All procedures performed were in accordance with the ethical standards of the 1964 Helsinki declaration and its later amendments.

### Case description

**Fig. 1:**
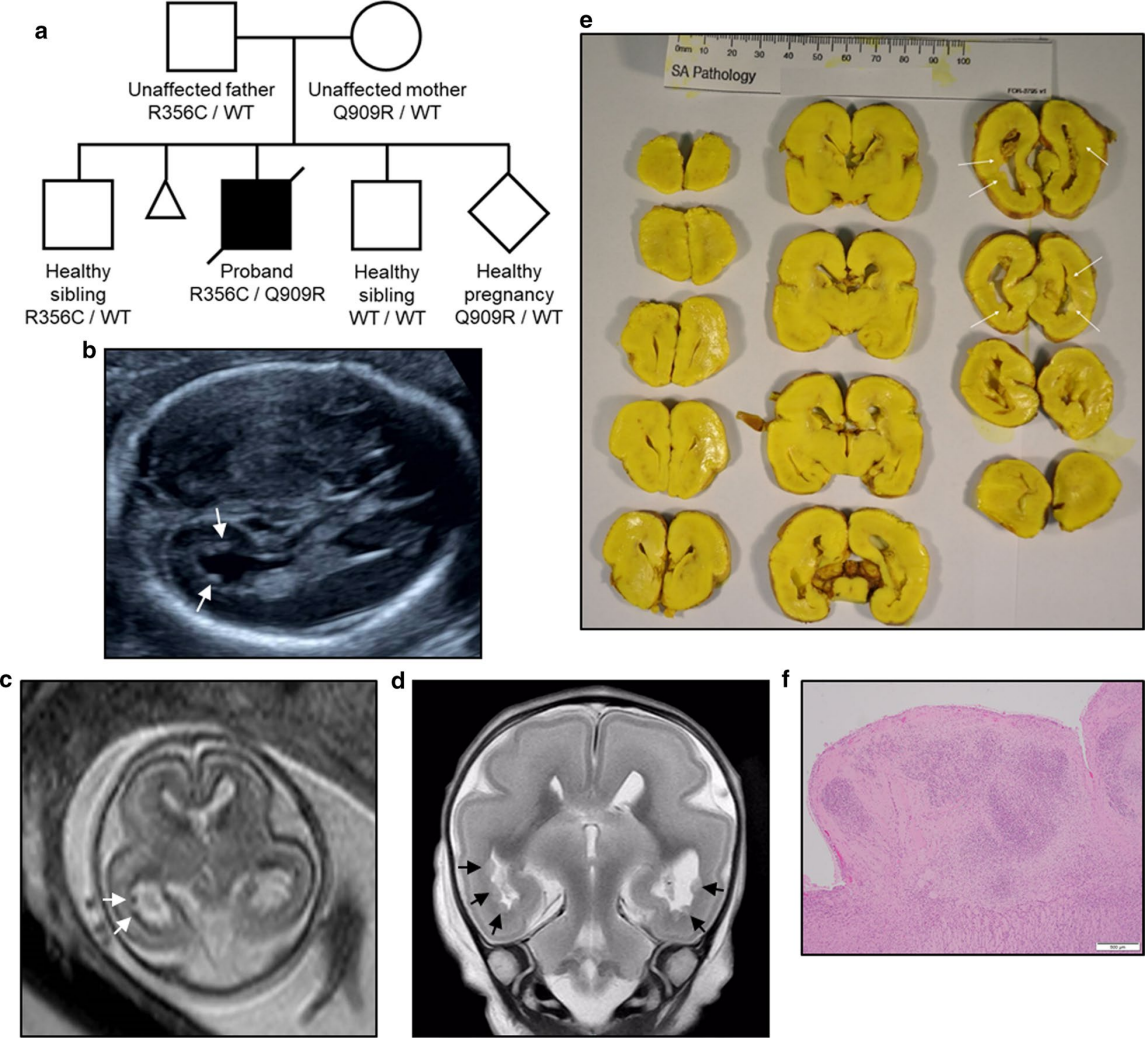
Neurological phenotype of a family with biallelic missense variants in *LAMC3*. **a** Pedigree of the non-consanguineous family. **b** Morphology ultrasound at 19 + 4 weeks gestation showing unilateral ventricular dilatation and irregularity of the cerebral ventricular wall (indicated by arrows). **c** Fetal MRI at 20 + 0 weeks gestation showing multiple foci of periventricular nodular heterotopia in the occipital lobes (indicated by arrows) [Axial T2 weighted image, 4 mm thick slices]. **d** Post mortem MRI at 21 + 5 weeks gestation shows extensive bilateral posterior periventricular heterotopia (indicated by arrows) [Coronal T2 weighted image, 2 mm thick slices]. **e** Coronal sections of the cerebrum (frontal top left to occipital bottom right). Macroscopically the brain shows normal gyration for gestation, with no evidence of polymicrogyria or loss of sulci usually visible at 21 weeks gestation to suggest early pachygyria. Subependymal nodularity is present within the occipital horns of the lateral ventricles (indicated by arrows). No further structural malformations of the brain are evident. **f** Microscopic image of a subependymal periventricular nodular heterotopia within the occipital horn of the lateral ventricle. The well circumscribed nodule is composed of disorganised primitive neurons within glioneuronal tissue. (Scale = 500 µm)

The parents of the male proband were non-consanguineous and healthy, with no known medical conditions. They have had 5 pregnancies; two healthy males, the proband, a healthy pregnancy currently in progress, and a history of one previous early miscarriage (Fig. 1a).

The proband pregnancy was conceived naturally and there were no early exposures to teratogens. Maternal health was good, although there was one episode of minimal vaginal bleeding at 6 weeks. The 12-week nuchal translucency scan was normal (NT 2.3 mm) and first trimester biochemical screening was low risk for trisomies 13, 18 and 21. The 19-week morphology ultrasound demonstrated unilateral ventricular dilatation and irregularity of the cerebral ventricular wall, suggestive of a possible diagnosis of PNH (Fig. 1b). The femur and humerus measured at the 10th centile, whilst the remainder of the long bones measured normal. No other abnormalities were seen. A fetal MRI performed at 20 + 0 weeks gestation showed left sided colpocephaly and irregularity of the fetal ependymal lining in the lateral ventricles, representative of subependymal nodular heterotopia (Fig. 1c). No other brain abnormalities were identified. Following the abnormal ultrasound results, a microarray (Illumina Infinium Global Screening Array-24v1.0, Illumina Inc, San Diego, CA, USA.) was performed on amniotic fluid cells. This confirmed a male fetus but no copy number variants (CNVs) were identified.

Based on the ultrasound findings, the couple elected for a termination of pregnancy at 21 + 5 weeks gestation and a perinatal autopsy was undertaken. The fetus weighed 440 g and growth parameters were consistent with a 21-week gestational age. Subtle dysmorphic features were apparent with a triangular face, widened anterior fontanelle and prominent heels. The remainder of the external examination was normal and there was no histological abnormality of the placenta. The internal thoracic and abdominal organs were normally formed and sited, and a radiological skeletal survey was normal. A post mortem MRI confirmed extensive PNH, predominantly involving the occipital horns of the lateral ventricles (Fig. 1d). The PNH was visible macroscopically at autopsy (Fig. 1e) and histology of affected tissue showed nodules of circumscribed glioneuronal tissue with disorganised, moderately dense aggregates of NeuN positive, primitive neurons (Fig. 1f, Additional file 1: Figure S1a). Some heterotopic nodules were also noted adjacent to the germinal matrix (Additional file 1: Figure S1b). No abnormalities of the corpus callosum were identified. The remainder of the cortex, brainstem and cerebellum were also macroscopically and histologically normal, with no evidence of polymicrogyria or pachygyria (Fig. 1e, Additional file 1: Figure S1c).

### Genetic analysis

Genomic DNA was isolated from whole blood (parents) or lung tissue (proband) and sequenced at the Broad Institute of MIT and Harvard’s Genomics Platform (Boston, MA, USA). Exonic regions were enriched using an Illumina exome capture (38 Mb target) and sequenced (150 bp paired reads) on an Illumina HiSeq (Illumina Inc, San Diego, CA, USA). Sequencing reads were aligned to the hg19 reference genome using BWA version 0.7.12, duplicate reads were removed and variants called using GATK HaplotypeCaller version 3.8.0. CNV’s were detected using an in-house (unpublished) algorithm, which partitions read depth signals into bin sizes optimal for the exome capture. CNV calling for the trio was performed in conjunction with 98 unrelated samples sequenced in the same batch, used to normalise read depth signals and remove sequencing-induced errors.

Single nucleotide variants (SNVs) were retained for being rare, with rare defined as ≤ 1% population frequency and ≤ 3 homozygous individual in gnomAD for autosomal or X-linked recessive inheritance, and ≤ 0.001% population frequency for autosomal dominant de novo inheritance [15]. Subsequent filtering selected for missense, truncating or canonical splice site protein-altering variants which were either called ‘de novo*’* or inherited in an X-linked or autosomal recessive manner from the unaffected parents. Using the same inheritance models, candidate CNVs were selected based on having no reciprocal overlap with known benign CNVs ≥ 70% in gnomAD, 1000G, or DGV, and being present in ≤ 2 unrelated samples from in-house data (n = 98). SNVs and CNVs were then prioritised based on the biological relevance of the affected gene to a neurological phenotype (OMIM, Ensembl).

## Results

A next-generation sequencing-based panel test, covering *ARFGE2, ERMARD, NEDD4L* and *FLNA* was performed in the clinical setting but no mutations were identified.

### Identification of candidate variants

Trio exome sequencing resulted in an average coverage of 83.56, with 89% of the exome target bases covered at least 20-fold (Additional file 4: Table S1). Variant calling provided 169,615 variants with an allele depth ≥ 5. Filtering for rare, protein altering variants revealed 7 variants in 4 genes considering autosomal recessive inheritance and 3 potential de novo variants (Additional file 4: Table S1). No significant copy number variants were identified. Further prioritisation of variants affecting genes associated with brain development indicated compound heterozygous variants in *LAMC3*: p.Arg356Cys (paternal; Chr9(GRCh37):g.133914340C>T, NM_006059.3:c.1066C>T,) and p.Gln909Arg (maternal; Chr9(GRCh37):g.133943597A>G, NM_006059.3:c.2726A>G,). Both *LAMC3* variants were confirmed by Sanger sequencing in the proband and parents, with further co-segregation analysis in the family revealing that an unaffected male sibling carried only the paternal variant (Fig. 1a, Additional file 2: Figure S2).

### Prenatal diagnosis

Following identification of the *LAMC3* variants, the parents elected for prenatal diagnostic testing, via chorionic villus sampling at 10-weeks gestation, in their two subsequent pregnancies. The first male pregnancy was found to not carry either variant, consistent with the normal 16-week ultrasound and normal morphology scan at 20-weeks. At birth there was no evidence of PNH and at 1 year the child is developing normally. The second pregnancy, currently at 28-weeks gestation, carries only the maternal variant, consistent with a normal 16-week ultrasound and normal morphology scans (Fig. 1a, Additional file 2: Figure S2).

## Discussion and conclusions

Here we present a 21-week-old male fetus with posterior PNH located in the occipital horns of the lateral ventricles, associated with colpocephaly, in which compound heterozygous *LAMC3* variants were identified.

*LAMC3* is located on chromosome 9 and encodes the laminin subunit ɣ3. Laminins are extracellular matrix glycoproteins required for cell adhesion, differentiation and migration. They are made of an alpha, beta and gamma chain, with different combinations of the subunit variants combining to form the various laminin isoforms [16]. The laminin ɣ3 subunit is utilised in 3 laminin isoforms, two of which have only been observed to be expressed in the central nervous system [16].

In the developing human fetal brain, *LAMC3* is expressed in both the VZ and throughout the cortical plate, with greatest expression in the region of the temporal and occipital lobes. At the subcellular level, *LAMC3* expression is localized to the cell bodies and dendrites of pyramidal neurons [17]. This expression is similar to that observed in known PNH genes, *FLNA* and *ARFGEF2*, although *LAMC3* does not seem to show the same localisation within the VZ to neuroependymal progenitors [18]. The spatial and temporal expression pattern of *LAMC3* is therefore consistent with mutations in the gene potentially causing a PNH phenotype. The expression pattern of human *LAMC3* is however in contrast to that of mice, where expression is localized to the pial basement membrane and cerebral vasculature, as well as to the intermediate zone, and the marginal zone of the cortical plate [17, 19].

The functional importance of Lamc3 is suggested by a homozygous knockout mouse model which shows cortical lamination defects and reduced brain size compared to wild-type [19]. Lamb2-Lamc3 double null mice show profoundly abnormal lamination, likely resulting from a combination of disruptions to the integrity of the pial basement membrane, altered radial glial cell morphology and aberrant distribution of Cajal-Retzius cells [19]. While the double null mouse has a phenotype that is more severe than either single null alone, it is currently unclear what the individual contribution of Lamb2 and Lamc3 is to these functions. Additional insight into the function of LAMC3, comes from Lamc3 knockout mice which demonstrate significantly delayed retinal astrocyte migration before later resolution to wild-type-like distribution [20]. Lamb2-Lamc3 double null mice show severely halted migration which does not resolve, and is again more severe than observed in either null individually [20]. Further support for a functional role of LAMC3 in migration is given by a knockdown zebrafish model which demonstrates defects in rostral primary motor neuron migration [21]. This proposed function of LAMC3 in migration and cortical development is consistent with mutations in the gene resulting in a PNH phenotype through failed migration and subsequent terminal differentiation of a subset of progenitor cells within the VZ.

In humans, biallelic mutations in *LAMC3* are responsible for a rare autosomal recessive condition characterized by pachygyria and polymicrogyria restricted to the occipital lobes (MIM 614,115) [17]. To date, 11 individuals from 5 consanguineous families have been described, with both truncating mutations and missense mutations reported [17, 22, 23]. In addition to the occipital cortical gyration abnormalities, affected individuals typically present with seizures (11/11 reported cases, all childhood onset where noted), and mild to moderate developmental delay (8/10 reported cases, not specified in 1 case) [17, 22, 23]. In one individual, while the pachygyria remained confined to the occipital lobe, the polymicrogyria extended to the frontal, parietal and temporal lobes of both hemispheres [23]. Although PNH has not been observed in any of the reported *LAMC3*-related cases, the predominantly occipital location of their MCD is consistent with the variants identified in our proband being the cause of the occipital restricted PNH.

Both of the *LAMC3* variants identified in our proband, p.Arg356Cys (94 alleles, 0.03%) and p.Gln909Arg (1 allele, 0.0004%), are rare in the population database gnomAD and have never been observed in homozygosity [15]. An alternate variant at amino acid 356, p.Arg356His, has been observed in homozygosity once, but the physicochemical properties of histidine are more similar to arginine than cysteine. Both variants are highly conserved (Additional file 3: Figure S3a and b) and computational pathogenicity prediction tools suggest the variants to have a deleterious effect (CADD: p.Arg356Cys: 26; p.Gln909Arg: 23). One pathogenic *LAMC3* missense variant has been previously reported, p.Gly350Arg (VCV000030419.1), which is located only 6 amino acids upstream from the p.Arg356Cys variant in our individual and within the same laminin-type EGF-like (LE) domain [20]. The introduction of a new cysteine at amino acid 356 may alter the disulfide bonding pattern, interfering with the highly conserved structure of the region and potentially affecting laminin-laminin interactions (Additional file 2: Figure S2) [24]. The p.Gln909Arg variant is also located in a LE domain in a region shown to be involved in nitrogen binding, an interaction important for basement membrane assembly [25].

This is the first report of biallelic *LAMC3* variants in a non-consanguineous family, and the first clinical association with posterior PNH, potentially broadening the phenotype of LAMC3-related disease. To our knowledge, this case is also the first to identify PNH on morphology ultrasound and MRI at such an early gestation. Our observation of this early phenotype should help in further elucidating the function of LAMC3 and its role in cortical development. For this family, exploratory exome analysis and the identification of likely causative variants facilitated prenatal diagnostic testing which provided reassurance in their two subsequent pregnancies.

## Supplementary Information

**Additional file 1: Figure S1**. Post mortem neurological findings. **a** NeuN positive nuclear labelling of primitive neurons within the heterotopic nodules. (Scale = 100 µm) **b** Thickening of the richly vascular germinal matrix with an adjacent heterotopic nodule. (Scale = 500 µm) **c** Cortical mantle showing normal layering and normal overlying meninges. (Scale = 500 µm).

**Additional file 2: Figure S2**. Family pedigree and segregation of the LAMC3 variants by Sanger sequencing. Both LAMC3 variants were confirmed to be present in compound heterozygosity in the proband. The paternal variant, p.Arg356Cys, and the maternal variant, p.Gln909Arg, were confirmed as heterozygous. Only the paternal variant was present in unaffected sibling 1, and only the maternal variant was present in the current unaffected pregnancy. The third unaffected sibling carries neither variant.

**Additional file 3: Figure S3**. Conservation of the LAMC3 variants. **a** The arginine residue at the location of the p.Arg356Cys variant (red box) is highly conserved across 100 species. The introduction of a cysteine at amino acid 356 may disrupt the completely conserved disulphide binding pattern of cysteines (blue boxes) in the region. **b** The glutamine residue at the location of the p.Gln909Arg variant (red box) is highly conserved across 100 species.

**Additional file 4: Table 1**. Exome sequencing results and variant filtering outcomes.

## Data Availability

Sequence data has been deposited at the European Genome-phenome Archive (EGA), which is hosted by the European Bioinformatics Institute and the Centre for Genomic Regulation, under accession number EGAS00001004793. Sequence variations and clinical assertions have been submitted to ClinVar (VCV000691960.2, VCV000691961.1). All unique materials and datasets generated and/or analysed during the current study are available from the corresponding author on reasonable request.

## Abbreviations

CNV: Copy number variant
HREC: Human Research Ethics Committee
LE: Laminin-type EGF-like
MRI: Magnetic resonance imaging
MCD: Malformations of cortical development
MIT: Massachusetts Institute of Technology
NHMRC: National Health and Medical Research Council
NT: Nuchal translucency
OMIM: Online Mendelian inheritance in man
PNH: Periventricular nodular heterotopia
SNVs: Single nucleotide variants
VZ: Ventricular zone
WCHN: Women's and Children's Health Network
NT: Nuchal translucency
