# Supplementary figures and images for: Compound heterozygous variants in LAMC3 in association with posterior periventricular nodular heterotopia
Source: BMC Med Genomics. 2021 Feb 27;14:64. doi: 10.1186/s12920-021-00911-4 (PMC7916305; doi:10.1186/s12920-021-00911-4)

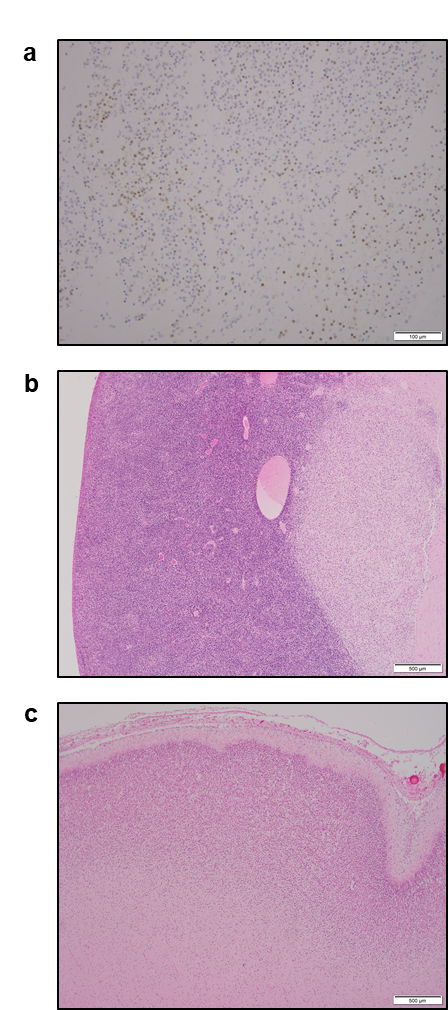

Supplement: Supplementary file 1 — Additional file 1: Figure S1. Post mortem neurological findings. a NeuN positive nuclear labelling of primitive neurons within the heterotopic nodules. (Scale = 100 µm) b Thickening of the richly vascular germinal matrix with an adjacent heterotopic nodule. (Scale = 500 µm) c Cortical mantle showing normal layering and normal overlying meninges. (Scale = 500 µm). [file 12920_2021_911_MOESM1_ESM.docx]

**
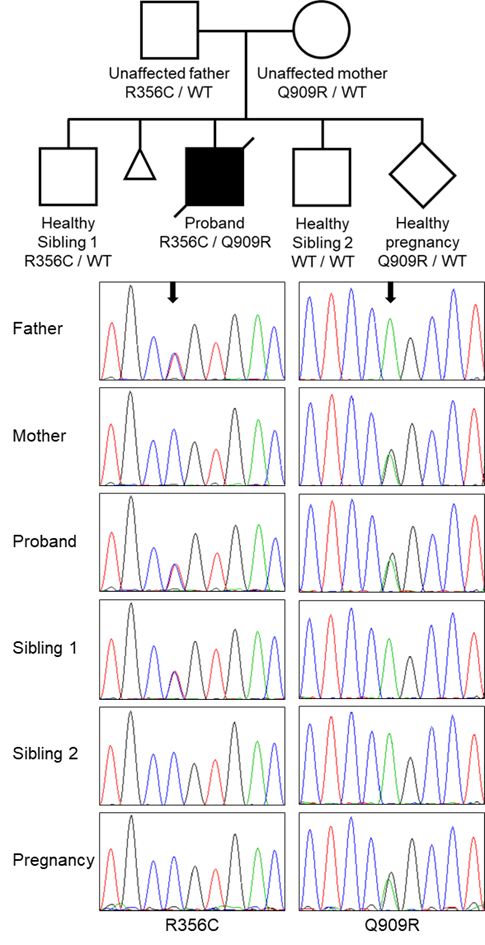
**

Supplement: Supplementary file 2 — Additional file 2: Figure S2. Family pedigree and segregation of the LAMC3 variants by Sanger sequencing. Both LAMC3 variants were confirmed to be present in compound heterozygosity in the proband. The paternal variant, p.Arg356Cys, and the maternal variant, p.Gln909Arg, were confirmed as heterozygous. Only the paternal variant was present in unaffected sibling 1, and only the maternal variant was present in the current unaffected pregnancy. The third unaffected sibling carries neither variant. [file 12920_2021_911_MOESM2_ESM.docx]

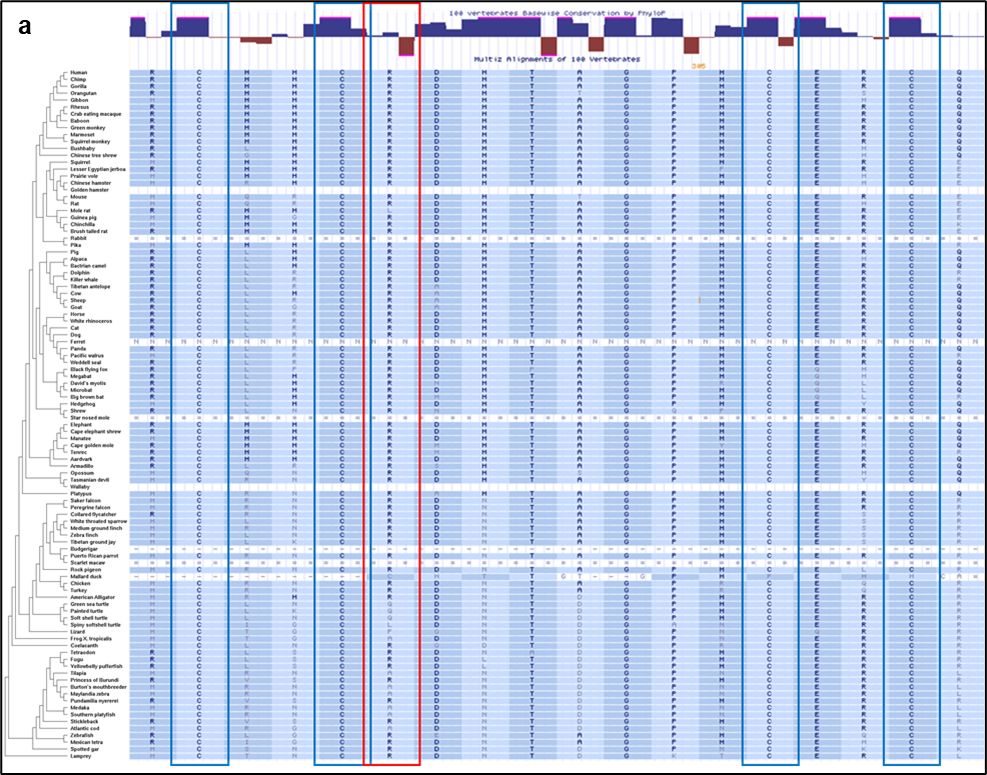


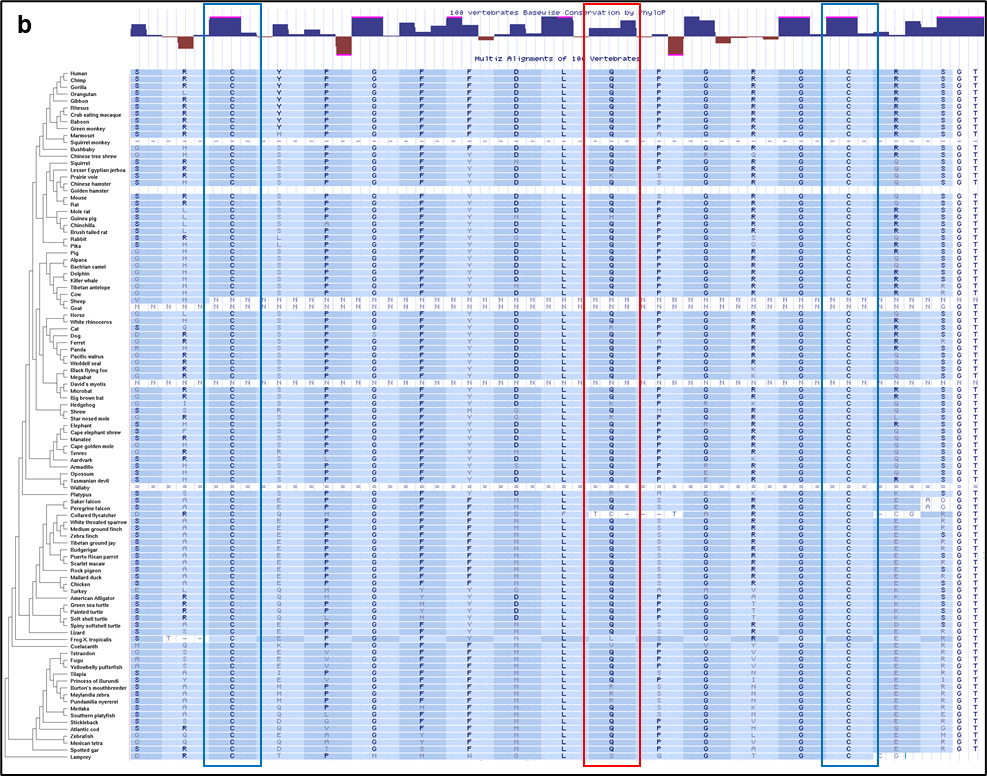

Supplement: Supplementary file 3 — Additional file 3: Figure S3. Conservation of the LAMC3 variants. a The arginine residue at the location of the p.Arg356Cys variant (red box) is highly conserved across 100 species. The introduction of a cysteine at amino acid 356 may disrupt the completely conserved disulphide binding pattern of cysteines (blue boxes) in the region. b The glutamine residue at the location of the p.Gln909Arg variant (red box) is highly conserved across 100 species. [file 12920_2021_911_MOESM3_ESM.docx]
