# Supplementary material for: Compound heterozygous variants in LAMC3 in association with posterior periventricular nodular heterotopia
Source: BMC Med Genomics. 2021 Feb 27;14:64. doi: 10.1186/s12920-021-00911-4 (PMC7916305; doi:10.1186/s12920-021-00911-4)
Supplement: Supplementary file 4 — Additional file 4: Table 1. Exome sequencing results and variant filtering outcomes. [file 12920_2021_911_MOESM4_ESM.docx]

**SUPPLEMENTARY TABLE**

**Supplementary Table 1: Exome sequencing results and variant filtering outcomes**

|  | **Proband** | **Mother** | **Father** | **Average** |
| --- | --- | --- | --- | --- |
| Sequenced gigabases (Gb) | 4.31 | 3.08 | 4.28 | 3.89 |
| Mean coverage (X) | 92.57 | 66.16 | 91.95 | 83.56 |
| % bases ≥ 20X | 89.6 | 87.6 | 90.3 | 89.17 |
|  |  |  |  |  |
| Total variants  (allele depth >5) | 169,615 |  |  |  |
| Rare variants  (≤1% population and ≤3 homozygous individual in databases) | 2,100 |  |  |  |
| Missense, truncating or canonical splice site variants | 267 |  |  |  |
| Variants under AR inheritance (homozygous or compound heterozygous) | 7 variants (4 genes)  SLC26A9 (Asn501Ser)  TMEM171 (Ser40Phe, Pro223Leufs*123)  LAMC3 (Arg356Cys, Gln909Arg)  ANKFN1 (Arg643His, Val942Ile) | | | |
| Variants under AD inheritance (possible *de novo*) | 3 variants (3 genes)  TRPS1 (Gly906Arg)  ZBTB3 (c.-52+1G>C)  SYNM (Arg103His) | | | |
| Variants in genes associated with neurological development | 2 variants (1 gene)  LAMC3 (Arg356Cys, Gln909Arg) | | | |
